# Morphological Variation and Spatial Distribution Patterns of *Krascheninnikovia compacta* (Losinsk.) Grubov in the Tibetan Antelope Breeding Grounds of the Western Kunlun Mountains

**DOI:** 10.3390/plants14091298

**Published:** 2025-04-25

**Authors:** Kailing Huang, Fengbing Lai, Mengyu Chen, Ying Song, Shujiang Chen, Zubaydah Wubuaysan, Xiaopeng Zhuang

**Affiliations:** 1 College of Geographical Science and Tourism, Xinjiang Normal University; Urumqi 830054, China; huangkailing839@gmail.com (K.H.); songying2001415@163.com (Y.S.); csjxjsfdx@163.com (S.C.); 15026284957@163.com (Z.W.); 188850267097@163.com (X.Z.); 2 Xinjiang Key Laboratory of Arid Zone Lake Environment and Resources, Urumqi 830054, China; 3 Beijing Key Laboratory of Precision Forestry, College of Forestry, Beijing Forestry University, Beijing 100083, China; cheny1992@bjfu.edu.cn

**Keywords:** western Kunlun Tibetan antelope breeding grounds, *Krascheninnikovia compacta*, morphological variation, spatial distribution, MaxEnt model

## Abstract

The study aims to analyze morphological variations and spatial distribution patterns of *Krascheninnikovia compacta* (Losinsk.) Grubov communities across 12 sampling areas at different elevations in the Tibetan antelope breeding grounds of the western Kunlun Mountains. Additionally, it projected the future climatically suitabie habitats of *K. compacta* under climate change scenarios, aiming to elucidate its community characteristics, spatial distribution dynamics, and the impacts of global warming on its growth. Integrated GIS, remote sensing, and unmanned aerial vehicles (UAVs) were used to investigate *K. compacta* communities. The Pearson correlation analysis revealed significant correlations between crown diameter, as well as between plant height and environmental factors. The redundancy analysis (RDA) results indicated that multiple environmental factors jointly explained the variation in plant height and crown diameter of *K. compacta*. Point pattern analysis, using the g(r) function combined with two null models, demonstrated changes in plant distribution during scale transitions. Additionally, the MaxEnt model was employed to project the potential suitable habitats of *K. compacta* under future climate scenarios. Overall, as the elevational gradient increases, the plant height of *K. compacta* gradually decreases while the crown diameter expands. Mean annual temperature (MAT) dominates the morphological variations in crown diameter and plant height, with lower temperatures correlating to shorter plant height and larger crown diameter. The complete spatial randomness (CSR) model indicates that across all elevations, the distribution patterns of plants transition sequentially from uniform to random, then clustered, and back to random as spatial scale increases. The heterogeneous Poisson (HP) model suggests that habitat heterogeneity is the primary driver of shifts in plant distribution patterns at larger scales. The MaxEnt model revealed distinct changes in suitable habitat areas of *K. compacta* under future climate scenarios. During 2061 to 2080s, its suitable habitats under the SSP126 and SSP585 pathways significantly contracted and expanded markedly, respectively.

## 1. Introduction

Plant height, a simple yet pivotal functional trait in plant ecological strategies, plays a critical role in regulating water balance, light interception, and carbohydrate transport, thereby influencing survival adaptation. As a predictor of species and ecosystem functionality, plant height reflects the dynamic equilibrium between species richness and environmental stress responses [1]. Studies have demonstrated that plant height determines species’ geographic distribution ranges by mediating ecological and evolutionary processes [2]. The canopy, through physiological activities such as photosynthesis, respiration, and transpiration, profoundly affects plant growth and development [3]. Crown diameter, a key indicator of canopy dimensions, serves as a proxy for plant health and productivity [4], with its size and shape holding significant ecological implications [5]. Morphological analyses of crown diameter and plant height can elucidate survival strategies under environmental gradients, revealing adaptive evolutionary mechanisms of biological communities at ecosystem scales [6]. Spatial distribution patterns (random, uniform, or aggregated) are essential for deciphering intra-specific interactions and plant–environment relationships [7]. Random distribution indicates the absence of distinct ecological interactions among communities; uniform distribution suggests negative ecological relationships (e.g., competitive exclusion) between communities; and aggregated distribution demonstrates positive ecological interactions within populations [8]. Climate change impacts ecosystems, and global warming causes alpine plant communities to shift towards higher elevation, potentially leading to the extinction of some unique plant groups [9]. In the context of global warming, mapping ecological bitmaps to predict changes in the potential suitable habitats of endangered plants is crucial for monitoring, managing, and restoring the increasingly dwindling species and habitats [10]. As a niche model, MaxEnt can predict the potential distribution of species based on a relatively small number of known species distribution points. It can be applied to endangered species, invasive species, forest destruction, etc. With a wide range of applications, it provides important references for formulating biodiversity conservation policies [11].

The western Kunlun Mountains in Xinjiang, China, constitute a critical breeding ground for the Tibetan antelope (*Pantholops hodgsonii*), hosting approximately 5000 individuals annually from mid-May to mid-July, primarily from the western population of the Qiangtang region [12,13]. As an endemic and endangered species of the Qinghai-Tibet Plateau, Tibetan antelopes inhabit alpine deserts at elevations of 3700 to 5500 m, with their migration patterns drawing extensive scientific attention [14]. *Krascheninnikovia compacta* (Losinsk.) Grubov, a cushion-forming dwarf semi-shrub of the genus *Krascheninnikovia* (Amaranthaceae), serves as the primary food source for Tibetan antelopes (*Pantholops hodgsonii*), accounting for over 50% of their diet [15] and providing essential nutrients. This species, characterized by its compact cushion-like morphology and typical height of 10–25 cm, exhibits remarkable ecological plasticity, tolerating extreme cold, drought, nutrient deprivation [16], and salinity [17]. It predominantly inhabits slopes and gravel terrains at elevations of 3500 to 5000 m in the Qilian Mountains (Gansu), Qinghai, Xinjiang, and Tibet, with sparse populations in the eastern Pamir region [18]. Notably, its high nutritional value and palatability make it a critical forage resource for both livestock and wildlife [19]. Despite its ecological and nutritional significance, existing research on *K. compacta* has focused predominantly on phylogenetics [20], environmental indicator functions [21], and genomic traits [22], leaving a knowledge gap regarding the synergistic mechanisms between its morphology (height/crown diameter) and spatial distribution patterns.

Therefore, a comprehensive understanding of morphological variations and spatial distribution patterns in *K. compacta* holds significant implications for deciphering local climatic adaptations, mitigating soil salinization, improving grassland management, combating desertification, and sustaining ecological stability—particularly in elucidating its relationship with Tibetan antelope breeding habitats. To address these objectives, this study employed Pearson correlation analysis to investigate morphological traits (plant height and crown diameter) in response to key environmental factors. Simultaneously, redundancy analysis (RDA) was employed to examine the responses of the morphological traits of *K. compacta* to natural environmental factors. Subsequently, point pattern analysis combined with null models was used to clarify the spatial distribution pattern. Finally, the MaxEnt model was utilized to predict the potential suitable habitat distribution of *K. compacta* under future climate conditions. This study aims to provide scientific evidence for ecological protection, ecological restoration, and biodiversity conservation in the Tibetan antelope breeding grounds in the Western Kunlun Mountains. In particular, it clarifies the community characteristics and spatial distribution patterns of *K. compacta*, a special species, and offers a theoretical basis for predicting the responses of alpine ecosystems to future climate change.

## 2. Results

### 2.1. Statistical Analysis of Plant Height and Crown Diameter

**Figure 1:**
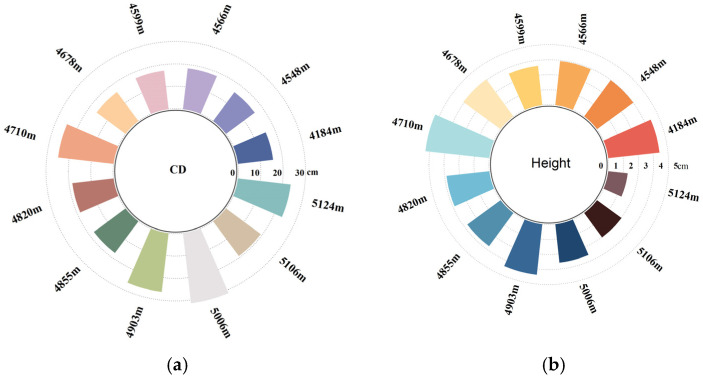
(**a**)The relationship between crown diameter and elevation; (**b**) The relationship between plant height and elevation.

Morphological statistics of *K. compacta* across 12 sample areas (Table 1 and Table 2) revealed significant dwarfing compared to other *Krascheninnikovia* species. *Krascheninnikovia arborescens* (Losinsk.) Czerep. in Shanxi, Inner Mongolia, and Hebei provinces typically reaches 1 to 2 m in height, while *Krascheninnikovia eversmanniana* (Stschegl. ex Losinsk.) Grubov in Xinjiang (Altai and Tianshan foothills) grows to 1–1.5 m. In contrast, the mean plant height of *K. compacta* in this study ranged merely from 1.3 to 4.29 cm, exhibiting a distinct trend of decreasing height and increasing crown diameter with elevation (Figure 1). The coefficient of variation (CV), a statistical measure of trait dispersion [23], was categorized as follows: CV ≤ 10% (highly stable), 10% < CV ≤ 20% (stable), 20% < CV ≤ 30% (unstable), and CV ≥ 30% (highly unstable) [24]. Plant height displayed overall instability (mean CV = 21.41%), with S11 showing extreme variability (CV = 37.3%) and the highest plasticity index (0.79), suggesting amplified environmental sensitivity at higher elevations. Conversely, crown diameter demonstrated relative stability (mean CV = 18.84%), though S10 exhibited the highest CV (31.35%), and S9 had the highest plasticity index (0.69). The mean plasticity indices (PI) for crown diameter (0.54) and plant height (0.60) further indicated that plant height responds more sensitively to environmental fluctuations. Kurtosis values for both traits were predominantly negative (indicating platykurtic distributions), while skewness values were mostly positive (right-skewed asymmetry). The geographical information of the 12 sample areas is detailed in the Table 3.

### 2.2. Morphological Changes of K. compacta

#### 2.2.1. Correlation Between Crown Diameter and Environmental Factors

**Figure 2:**
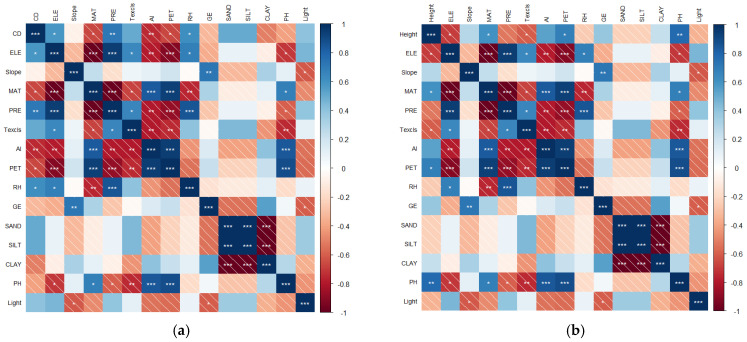
(**a**) Thermographic correlation matrix between crown diameter and environmental factors; (**b**) Thermographic correlation matrix between plant height and environmental factors. CD represents crown diameter (cm), CD denotes plant height (cm), ELE indicates elevation (m), and slope refers to slope gradient (°). Climatic variables include MAT, which represents mean annual temperature (°C); PRE represents precipitation (mm); AI represents aridity index aridity index; and PET represents potential evapotranspiration (mm). RH represents relative humidity (%), and light represents solar radiation (WH/m^2^) characterizing atmospheric conditions. Texcls soil texture denotes classification by the USDA system, SANDS indicates sand content (0 to 30 cm, %), CLAY indicates clay content (0 to 30 cm, %), SILT indicates silt content (0 to 30 cm, %), and pH indicates soil acidity (15 to 30 cm). GE categorizes geomorphic types using genetic classification. Same as below. (* *p* < 0.05, ** *p* < 0.01, *** *p* < 0.001).

As shown in Figure 2a, crown diameter exhibited a significant increase with rising elevation (*p* < 0.05). Concurrently, crown diameter gradually enlarged as the mean annual temperature (MAT) showed a marked decline (*p* < 0.05). Precipitation (PRE) positively influenced crown diameter development, with greater rainfall correlating strongly with larger crown dimensions (*p* < 0.01), indicating its critical role in facilitating plant coverage expansion. Under drought stress conditions characterized by high aridity index (AI) and potential evapotranspiration (PET), *K. compacta* demonstrated reduced resource allocation to crown growth (*p* < 0.01 for AI; *p* < 0.05 for PET). Relative humidity (RH) acted as a growth-promoting factor for crown expansion (*p* < 0.05).

#### 2.2.2. Correlation Between Plant Height and Environmental Factors

As illustrated in Figure 2b, plant height exhibited a decreasing trend with ascending altitudinal gradient (*p* < 0.05). Under conditions of MAT increase (*p* < 0.05) and elevated PET (*p* < 0.05), plant height demonstrated significant positive responses. Furthermore, plant height displayed adaptive sensitivity to soil textural types (Texcls), showing increased vertical growth as soil texture transitioned toward sandy loam and sandy classifications (*p* < 0.05), indicative of phenotypic plasticity advantages. Notably, soil pH elevation exerted a strongly positive influence on plant height development (*p* < 0.01). Collectively, altitudinal gradient, MAT, Texcls, PET, and soil pH emerged as statistically significant environmental determinants governing plant height variation.

#### 2.2.3. Morphological Responses of Plant Height and Crown Diameter to Environmental Drivers

**Figure 3:**
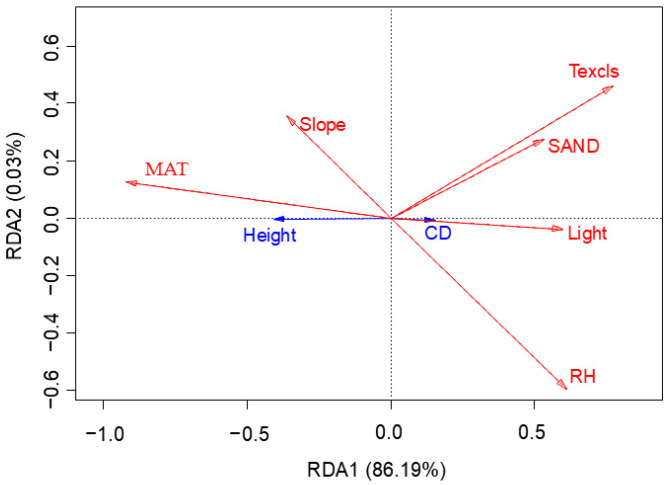
RDA of environmental and morphological factors in *Krascheninnikovia compacta* (Losinsk.) Grubov.

The overall model performance was statistically significant (*p* < 0.05), with the first axis of the RDA explaining a significant proportion of variance (*p* < 0.05), indicating robust structural constraints within the dataset. RDA triplot (Figure 3) revealed coordinated variations between plant height, crown diameter, and environmental drivers (red vectors: environmental factors; blue vectors: morphological traits). The first two axes collectively explained 86.22% of the variance (RDA 1: 86.19%, RDA 2: 0.03%). Multiple environmental factors synergistically shaped the morphological plasticity of *K. compacta* (Table 4), an endemic cushion plant in the Tibetan antelope *Pantholops hodgsonii* breeding grounds of the western Kunlun Mountains. This unique alpine environment has driven evolutionary adaptations in *K. compacta*’s architecture. Highly significant positive correlation (*p* < 0.001) with plant height shows dwarfing (reduced vertical growth) but expanded crown diameter under colder conditions. In summary, MAT exhibited additive interactions, jointly regulating the allometric balance between height and crown diameter investment.

### 2.3. Spatial Distribution Pattern of K. compacta

#### 2.3.1. Spatial Density of *K. compacta* Across Elevational Gradients

**Figure 4:**
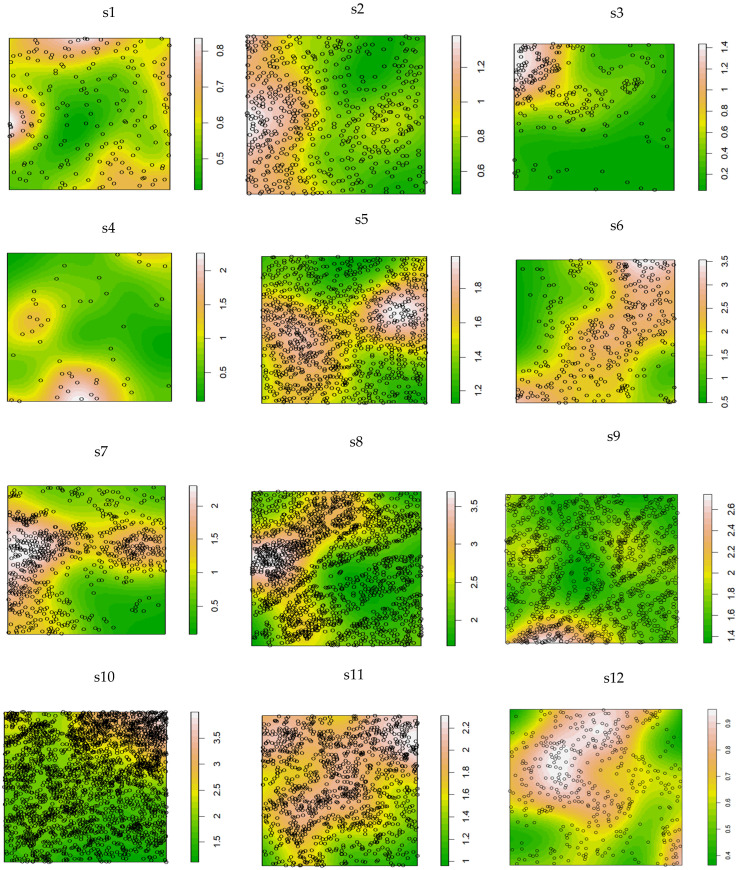
Distribution density of *K. compacta* in 12 subplots at different elevations. Note: s1–s12 correspond to S1–S12, representing the selected subplots.

As illustrated in Figure 4, the spatial density of *K. compacta* exhibited distinct regional clustering. The highest densities (3.5 ind./m^2^) were observed in subplots s6, s8, and s10. In s6, individuals were concentrated in a northeast-southwest oriented belt-like distribution. s8 harbored dense aggregations in its western sector, while s 10 showed clustering in the northeastern region. Lower densities were recorded in s1 (0.8 ind./m^2^) and s12 (0.9 ind./m^2^), which displayed contrasting spatial trends: s1 exhibited a radially decreasing density gradient from the periphery toward the center, whereas s12 showed a concentrically decreasing gradient from the center outward. The remaining sampling sites demonstrated heterogeneous distribution patterns. These findings suggest that elevational variations in *K. compacta* spatial density are likely influenced by localized environmental factors, resulting in site-specific distributional configurations across elevational gradients.

#### 2.3.2. Spatial Distribution Heterogeneity of *K. compacta* Across Elevational Gradients

**Figure 5:**
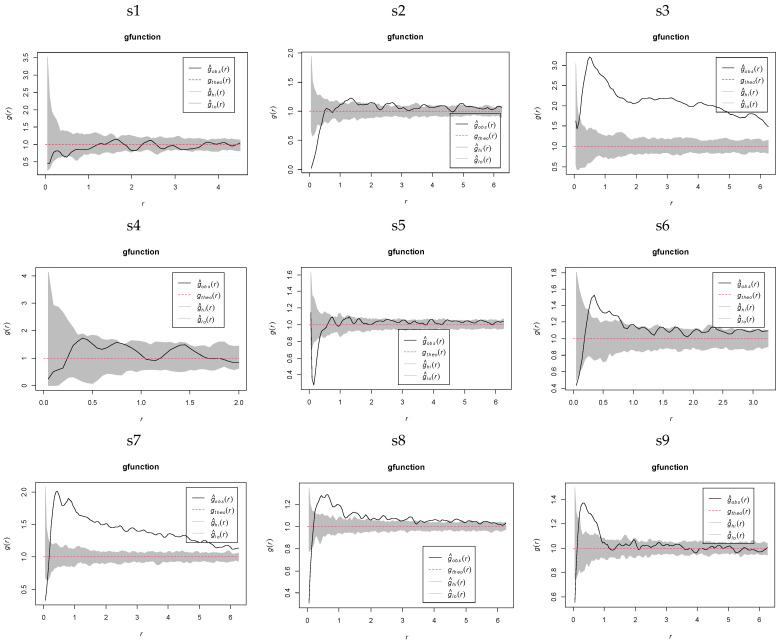

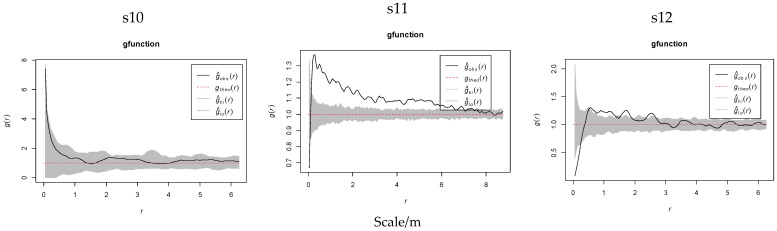
Spatial point pattern analysis of *K. compacta* populations at varying elevations under CSR. Note: In the figure, the gray shaded area represents the upper and lower envelope line region, which is the 95% confidence interval. The ${\hat{g}}_{obs}$(*r*) is the observed value of *g*(*r*), and ${\hat{g}}_{theo}$(*r*) is the theoretical value of *g*(*r*).The ${\hat{g}}_{hi}$(*r*) represents the upper envelope (curve), ${\hat{g}}_{lo}\left(r\right)$ represents the lower envelope (curve). Same as below.

Spatial point pattern analysis using the complete spatial randomness (CSR) model revealed scale-dependent distribution dynamics of *K. compacta* across elevational gradients (Figure 5). Populations at s1, s4, and s10 exhibited random distributions across all spatial scales. In contrast, populations at s6, s8, s9, and s11 transitioned sequentially from uniform to random distributions at minimal scales, followed by clustered distributions at intermediate scales (s6: 0.2 to 0.85 m; s8: 0.3 to 4.85 m; s9: 0.2 to 0.65 m; s11: 0.24 to 7.1 m), ultimately reverting to randomness at larger scales. while s7 and s3 exhibited initial divergence in minimal-scale patterns but converged to clustering with increasing scales. Populations at s2 and s12 alternated between clustered and random distributions beyond 0.2 m, whereas s5 uniquely displayed a reverse transition from randomness to uniformity at 0.01 to 0.4 m, followed by extended randomness up to 6 m. These findings demonstrate that *K. compacta* exhibits a multi-phase spatial transition across elevational gradients: uniform → random → clustered distribution at small spatial scales, gradually shifting to random distribution with increasing spatial scales.

**Figure 6:**
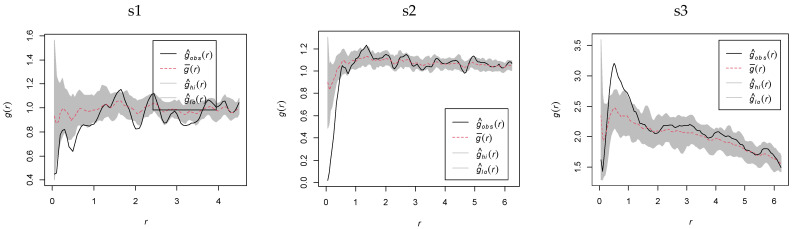

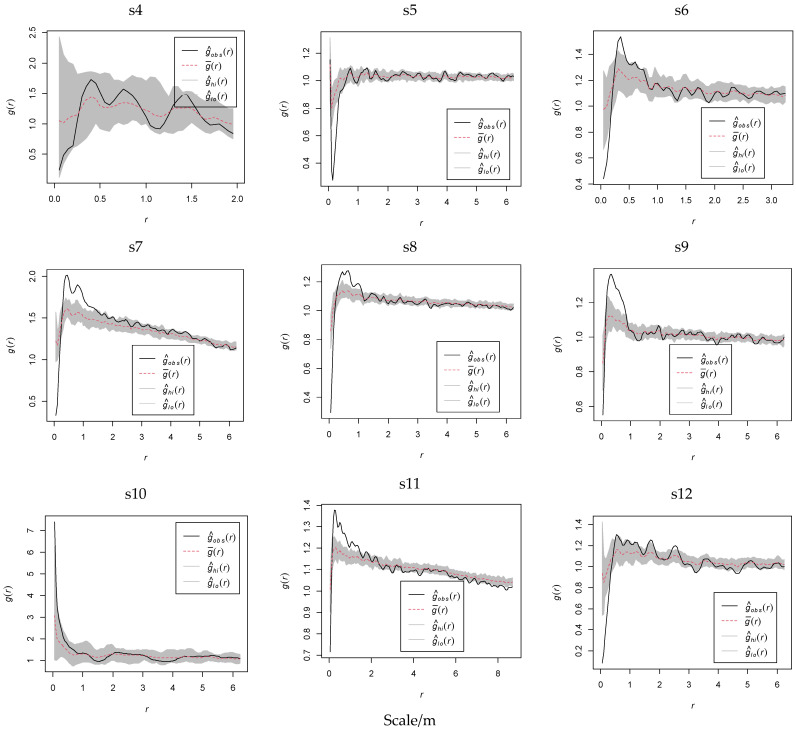
Spatial point pattern analysis of *K. compacta* populations at varying elevations under HP.

The heterogeneous Poisson (HP) model, accounting for habitat heterogeneity, revealed that populations of *K. compacta* across elevations predominantly transitioned from uniform → random → clustered → random distributions with increasing spatial scales (Figure 6). Compared to the CSR, the HP framework extended the spatial extent of random distributions. Subplots s4 and s10 conformed to the HP model across all scales, while clustered distributions in s3, s6, s7, s8, s9, and s11 were restricted to narrow ranges (s3: 0.3 to 0.9m; s6: 0.3 to 0.4 m; s7: 0.25 to 1.1 m; s8: 0.15 to 1.05 m; s9: 0.2 to 0.9 m; s11: 0.35 to 1.85 m). Sites s2, s5, and s12 exhibited uniform distributions at minimal scales (s2: 0 to 0.55 m; s5: 0.05 to 0.45 m; s12: 0 to 0.5 m), shifting to randomness under habitat heterogeneity. In contrast, s1 displayed alternating uniform-random patterns without clustering. These results highlight elevation-specific responses.

### 2.4. MaxEnt Model Analysis

#### 2.4.1. MaxEnt Model Optimization and Parameter Evaluation

**Figure 7:**
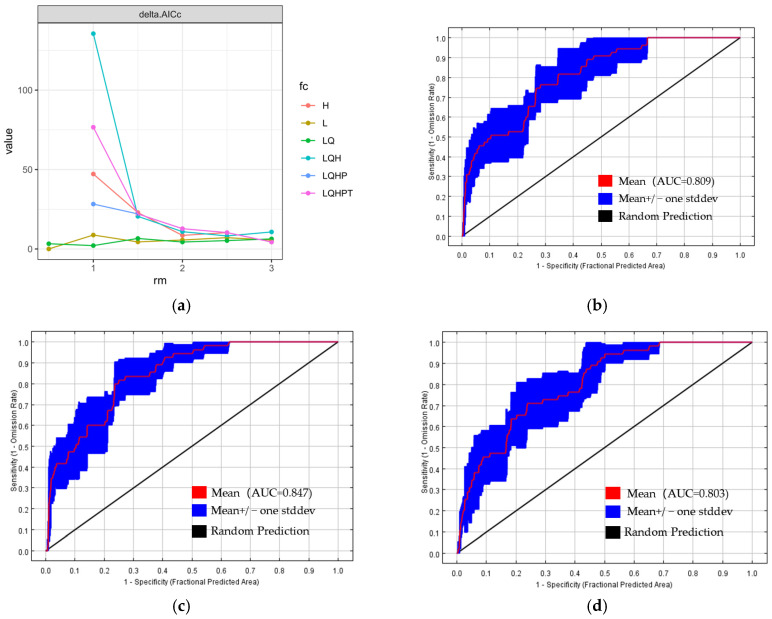
ΔAICc plot and time-period-specific ROC curves. (**a**) R-tuned ΔAICc plot; (**b**) ROC curve under current climate condition; (**c**) ROC curve under SSP126-2061 to 2080s; (**d**) ROC curve under SSP585-2061 to 2080s.

See Figure 7 for details; based on cross-validation of species data and environmental factors across feature combinations (FC) and regularization multipliers (RM), the optimal parameters were selected as FC = L (linear) and RM = 0.5. Under current climatic conditions, the mean test AUC value was 0.809 with a standard deviation (SD) of 0.061. For the SSP126 scenario (2061 to 2080s), the mean test AUC value was 0.847 (SD = 0.042), while under the SSP585 scenario (2061 to 2080s), it reached 0.803 (SD = 0.047). These results indicate robust model performance with good accuracy and high predictive capability.

#### 2.4.2. Current and Future Habitat Distribution

**Figure 8:**
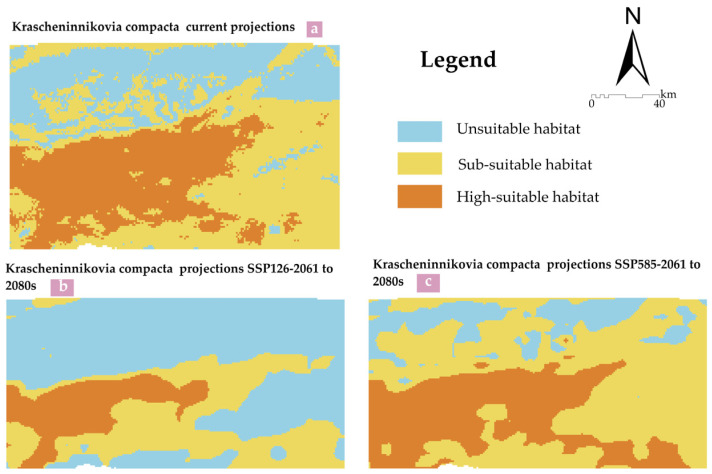
The distribution of suitable habitat areas for *K. compacta* under current and future climate conditions. (**a**) The distribution of suitable habitat areas for *K. compacta* under the current climate; (**b**) The distribution of suitable habitat areas for *K. compacta* under the SSP126 pathway from 2061 to 2080 in the future climate; (**c**) The distribution of suitable habitat areas for *K. compacta* under the SSP585 pathway from 2061 to 2080 in the future climate.

To analyze the future climate-driven changes in the suitable habitat areas of *K. compacta*, the MaxEnt model outputs revealed that the mean MTSS and TPT values of the trained model were 0.3731 and 0.1937, respectively, which were used as thresholds to reclassify the model results (Figure 8). Statistical results indicate that under current climate conditions, the areas of highly suitable habitat, sub-suitable habitat, and unsuitable habitat for *K. compacta* are 0.43 × 10^4^ km^2^, 0.65 × 10^4^ km^2^, and 0.39 × 10^4^ km^2^, accounting for 29.28%, 44.19%, and 26.53% of the total study area, respectively (Table 5). The total suitable habitat area (73.47% of the study area) suggests that the region is generally favorable for *K. compacta* growth.

Under future climate scenarios, habitat suitability shifts significantly. For the SSP126 pathway (2061 to 2080s), highly suitable habitat, sub-suitable habitat, and unsuitable habitat areas are 0.2 × 10^4^ km^2^ (13.48%), 0.44 × 10^4^ km^2^ (29.84%), and 0.83 × 10^4^ km^2^ (56.67%), respectively. In contrast, under the SSP585 pathway (2061 to 2080s), these values change to 0.44 × 10^4^ km^2^ (30.26%), 0.81 × 10^4^ km^2^ (55.6%), and 0.21 × 10^4^ km^2^ (14.14%). Compared to the current climate, the SSP126 pathway shows a substantial reduction in suitable habitat area, with highly and sub-suitable areas decreasing by 53.49% and 32.3%, respectively. Conversely, under the SSP585 pathway, suitable habitat areas expand, with highly and sub-suitable areas increasing by 2.33% and 24.6%, respectively.

**Figure 9:**
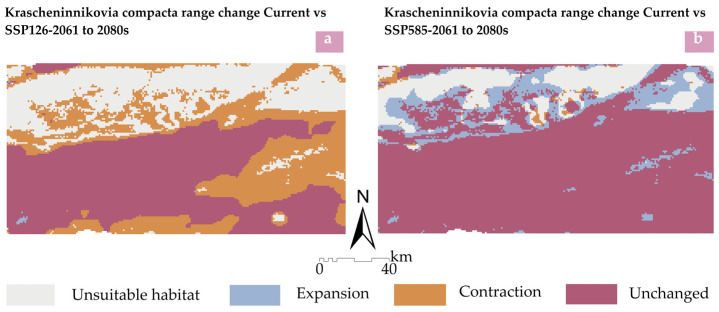
Distribution maps showing range change area of *K. compacta* under future climatic scenarios. (**a**) Comparison of suitable habitat areas for *K.compacta* between current (1970 to 2000s) and future (2061 to 2080s) under the SSP126 pathway; (**b**) Comparison of suitable habitat areas for *K. compacta* between current and future (2061 to 2080s) under the SSP585 pathway.

As can be seen from Figure 9 and Table 6, under the future SSP126 pathway, from the current period to 2061 to 2080s, the potential suitable habitat areas for *K. compacta* generally show a shrinking trend. The shrinking area of the suitable habitat accounts for 30.26% of the total study area, while the proportion of the expanded area only accounts for 0.1%, and the area of the unchanged region accounts for 43.21%. The reduction in the suitable habitat areas is mainly concentrated in the northern, eastern, and southern parts. Under the future SSP585 pathway, from the current period to 2061 to 2080s, the potential suitable habitat areas for *K. compacta* generally show an expanding trend. The expanded area accounts for 13.4% of the total area, the reduced area accounts for 1% of the total area, and the unchanged region accounts for 72.46% of the total study area.

## 3. Discussion

### 3.1. Adaptive Morphological Plasticity in Plant Height and Crown Diameter of K. compacta

Plant phenotypic plasticity, defined as the capacity to develop variable phenotypes in response to environmental conditions, represents a critical adaptive mechanism for surviving climate change and global change drivers [25]. In this study, correlation analyses revealed that individual traits exhibited significant associations with multiple environmental factors, indicating that morphological characteristics of *K. compacta* arise from the integrated effects of various abiotic drivers. While Pearson correlations can identify relationships between single traits (e.g., crown diameter or plant height) and specific environmental variables, focusing on isolated traits fails to capture the holistic evolutionary strategies plants employ to adapt to complex environmental gradients, which typically involve trade-offs and synergies among multiple morphological traits [26]. Integrated analysis of height-crown allometry and environmental factors demonstrates that *K. compacta’s* compact, prostrate cushion morphology—a hallmark adaptation to extreme alpine cold—is primarily driven by low-temperature stress rather than aridity [27]. This architectural strategy optimizes resource allocation: reduced vertical growth minimizes exposure to freezing winds and radiative heat loss, while expanded crown enhances light interception and thermal buffering. Such structural adaptations mitigate cellular damage and physiological disruptions, enabling functional recovery under harsh conditions (e.g., subzero temperatures, high winds, intense radiation) [28]. Furthermore, cushion microclimates elevate internal temperatures by 5 to 9 °C compared to ambient air [29]. This morphological plasticity reflects the resource allocation strategies of alpine plants across stress gradients, dynamically balancing environmental stressors through vertical contraction and horizontal expansion. Through their study of cushion plants in the Himalayas, Rasray et al. discovered [30] that these foundational species in alpine communities play a crucial role in maintaining biodiversity. This is primarily manifested in their ability to ameliorate local microenvironmental conditions, such as soil nutrient availability, temperature regulation, and moisture retention. By modifying these ecological parameters, cushion plants create favorable growing conditions for neighboring plant species through positive facilitative interactions. This ecological engineering establishes cushion plants as climate refugia for plant diversity under environmental change, thereby serving as critical stabilizers in alpine ecosystem functioning and making essential contributions to the preservation of high-altitude ecological stability.

### 3.2. The Spatial Distribution Pattern of Crown Width and Plant Height in K. compacta

As the elevation gradient increases, various environmental factors such as temperature, precipitation, solar radiation, and wind speed undergo rapid changes over short distances, which directly or indirectly influence plant diversity, composition, and distribution [31]. The density and distribution of *K. compacta* exhibit varying degrees of alteration along the elevation gradient, indicating that endemic species communities at different elevations can adopt multiple ecological strategies to withstand harsh environmental stresses under specific conditions [32].

The spatial distribution patterns of plant populations result from interactions among intrinsic population characteristics, interspecific relationships, and environmental conditions [33], which are closely associated with habitat heterogeneity. With increasing elevation, habitat heterogeneity intensifies, leading to habitat fragmentation and subsequently affecting plant spatial distribution patterns [34]. The point pattern analysis in this study revealed distinct spatial patterns in *K. compacta* communities across altitudinal gradients. The research area, located on the Tibetan Plateau, features high elevation, frigid-arid climates, and low soil fungal diversity, which impair soil carbon-nitrogen cycling and plant mineral nutrient uptake, creating challenging conditions for vegetation growth [35]. Overall, *K. compacta* exhibited low density within sample plots, primarily showing uniform-random-aggregated-random distributions, consistent with patterns observed in other alpine ecosystems [36,37]. Some studies suggest that at small scales, spatial patterns are influenced by intraspecific competition, seed dispersal mechanisms, and habitat-specific adaptations [38]. *K. compacta* predominantly relies on clonal propagation (tillering) under harsh conditions, generating new ramets near maternal plants [39]. Limited dispersal efficiency often concentrates ramets around parent individuals, exacerbating interspecific competition for resources at close ranges [40], thereby manifesting uniform or random distributions at microscales. Outside the cushion, individuals may form aggregated patterns for mutual protection against extreme alpine stresses, though escalating competition for residual resources with increasing crown coverage eventually shifts distributions toward randomness. The random-aggregated (s3) and uniform-random-aggregated (s7) patterns observed in specific plots likely represent ecological strategies to mitigate small-scale competitive pressures while adapting to composite environmental constraints (topography, soil, moisture, light), ultimately favoring aggregated distributions at broader scales [41]. In sample plots s1, s4, and s10, random distributions across all scales may stem from animal trampling and grazing alongside intraspecific competition. Under the heterogeneous Poisson model, uniform or aggregated patterns at finer scales reflect competitive interactions or reproductive traits, yet all altitudinal populations conformed to this model at larger scales, emphasizing habitat heterogeneity as a critical driver of spatial patterns across scale gradients. Notably, in high-elevation zones, microenvironmental variability profoundly shapes species distribution features, generating complex biogeographic patterns [42]. Additionally, considering the influence of plant reproductive dispersal on spatial pattern formation, *K. compacta* exhibits clonal propagation traits. Similar to other desert plants, it relies on basal tillering—a vegetative reproduction strategy where new ramets emerge from the base of parent plants—to adapt to harsh environments [43].

### 3.3. Prediction of the Suitable Habitat Areas for K. compacta Using MaxEnt

Understanding the shifts in potential species distribution patterns under climate change is critical for evaluating its impacts on species and developing conservation strategies. As a dominant species adapted to high-altitude cold ecosystems, cushion plants exhibit specific distribution patterns under climate change. This study reveals that under current and future climatic conditions, the suitable habitats of *K. compacta* in the research area are concentrated in high-elevation zones, showing a decreasing trend from the periphery toward the central-western regions. However, habitat areas vary significantly across different climate pathways. Under the SSP126 scenario, the total suitable habitat area (including sub-suitable and highly suitable zones) during 2061 to 2080s is projected to be 0.63 × 10 km^2^, accounting for 43.32% of the study area—lower than unsuitable areas and representing a 30.26% reduction compared to current conditions. In contrast, under the SSP585 pathway, the suitable habitat area expands markedly to 1.26 × 10 km^2^ (85.86% of the total area) during the same period, with an expansion rate of 13.4% relative to current distributions. Previous studies indicate [44] that temperature increases enhance photochemical efficiency, water use efficiency, and reproductive capacity in Andean cushion species like *Azorella madreporica* (Apiaceae). Similarly, Pugnaire et al. demonstrated [45] that elevated temperatures improve photosynthesis and growth in Sierra Nevada’s cushion plant *Arenaria tetraquetra* ssp. *amabilis* (Caryophyllaceae). For *K. compacta*, warming drives upward elevational shifts, with asexual reproduction dominating in harsh environments. However, temperature rises may improve seed production (enhancing sexual reproduction), increase leaf carbon storage, and allocate biomass toward larger, thinner leaves and larger individual plants [46].

Uncontrolled temperature escalation could drastically alter species distributions, productivity, and interactions in high-elevation ecosystems. Cold-adapted species may migrate upward, while subalpine species encroach on their original habitats, potentially leading to habitat fragmentation, range contraction, and even extinction [47]. Therefore, maintaining stable temperatures is critical. Long-term monitoring of habitat dynamics, improved management of existing populations, mitigation of anthropogenic impacts, and implementation of in situ conservation measures with stringent protection policies are urgently needed to safeguard this unique ecotype.

## 4. Materials and Methods

### 4.1. Materials

#### 4.1.1. Study Area

**Figure 10:**
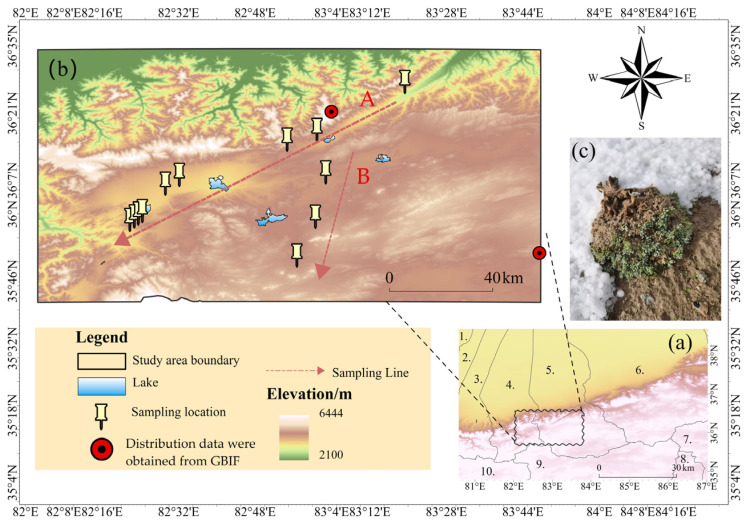
Geographical map of the research area. (**a**) The surrounding area of the research area; (**b**) The elevation of the research area; (**c**) *Krascheninnikovia compacta*. Note: **1.** Moyu County; **2.** Luopu County; **3.** Qira County; **4.** Yutian County; **5.** Minfeng County; **6.** Qiemo County; **7.** Shuanghu County; **8.** Nima County; **9.** Gêrzê County; **10.** Rutog County. A and B are the survey transect numbers.

The study area is located in the western Kunlun Mountains, a critical breeding ground for Tibetan antelopes (average elevation > 4600 m), bordered by the Ngari Prefecture of Tibet to the south, Minfeng County to the east, and Yutian County to the west. Its geographic coordinates span 82°55′7.424″–83°47′44.837″ E and 36°8′4.26″–37°7′15.623″ N, situated within the Tibetan Plateau. Characterized by a hyper-arid desert climate due to its inland remoteness, mountainous barriers, and limited moisture influx, the region receives minimal precipitation. The warmest month records only 14 to 20 mm of rainfall, with annual precipitation ranging from 54 to 95 mm. The coldest month averages temperatures of −21 to −17 °C, while the warmest month ranges from 5 to 9 °C, yielding an annual mean temperature of −18 to 11 °C (study area overview shown in Figure 10). Vegetation in this area predominantly exhibits a cushion-like morphology—low-growing, densely branched structures—an evolutionary adaptation to extreme alpine conditions, intense solar radiation, and persistent strong winds. Consequently, cushion plants dominate as the keystone ecological type in this high-elevation desert. Field surveys identified *K. compacta* as the dominant species, accompanied by sparse companion species such as *Androsace umbellata* (Lour.) Merr. and *Oxytropis microphylla* (Pall.) DC. [48]. Soils are primarily classified as cryoarid soils, desert soils, and calcic soils, with localized saline-alkali patches. 12 sample areas (Table 3) were established across elevation gradients of 4138 to 5114 m, targeting representative *K. compacta* habitats. Integrated unmanned aerial vehicle (UAV) remote sensing and ground-based surveys were employed to quantify morphological and distributional characteristics of *K. compacta* populations.

#### 4.1.2. Data

Given the minimal anthropogenic disturbance in the region, this study selected natural environmental factors relevant to *K. compacta* growth, including climate variables, solar radiation, topographic features, and soil properties. All data were sourced from publicly available databases: administrative boundaries from the. Geographic Data Sharing Infrastructure, global resource data cloud.(http://www.gis5g.com, accessed on 6 September 2024); a 250 m spatial resolution digital elevation model from the Geospatial Data Cloud (https://www.gscloud.cn/search, accessed on 1 April 2024), with slope and aspect derived via the ArcGIS 10.8 3D Analyst module; geomorphological data (1 km spatial resolution) and multi-year precipitation/mean annual temperature datasets (1960 to 2010s, 1 km spatial resolution) from the Resource and Environmental Science Data Platform (https://www.resdc.cn, accessed on 13 January 2025); soil texture (USDA classification) and pH (15 to 30 cm depth, 250 m spatial resolution) from the National Earth System Science Data Center (http://www.geodata.cn, accessed on 13 January 2025); relative humidity (2000 to 2020s, 1 km spatial resolution) from the same center; aridity index and potential evapotranspiration (2000 to 2020s, 1 km spatial resolution) from the National Tibetan Plateau Data Center (http://data.tpdc.ac.cn, accessed on 14 January 2025); solar radiation data calculated using the DEM through the “Area Solar Radiation” tool in ArcGIS 10.8; and soil sand, clay, and silt content (0 to 30 cm depth, 1 km spatial resolution) from the Harmonized World Soil Database v1.2 (https://www.fao.org/soils-portal, accessed on 15 September 2024). All raster datasets were uniformly resampled to 250 m × 250 m spatial resolution in ArcGIS 10.8, followed by multi-value extraction to the centroid coordinates of each study sample for integrated spatial analysis. In addition to the 12 field survey sample points, the remaining two distribution points of *K. compacta* used in the MaxEnt model analysis were sourced from the Global Biodiversity Information Facility (GBIF, www.gbif.org, accessed on 20 August 2024). The two points are located at 83°47′27.6″ E, 35°51′50.4″ N and 83°3′57.161″ E, 36°21′18.4″ N. Global warming necessitates enhanced assessment of plant species’ habitat suitability shifts under future climate regimes, and environmental data were obtained from Worldclim (https://www.worldclim.org, accessed on 21 August 2024), including 19 bioclimatic variables for the current (1970 to 2000s) and future (2061 to 2080s) periods. Future climate projections utilized the BCC-CSM2-MR global climate model, appropriate for China, under Shared Socioeconomic Pathways with low- and high-emission scenarios (SSP126 and SSP585). The 19 environmental variables, at a resolution of 30’ (approximately 1 km × 1 km), were extracted for the study area using a mask. All coordinates are based on WGS84.

#### 4.1.3. Sampling Areas Design

Based on preliminary route planning, field investigations were carried out in late July 2023 along two east-west transects (A and B) spanning an elevation gradient of 4138 to 5114 m. To ensure adequate image overlap rates and interpretation accuracy while minimizing challenges associated with establishing large plots, 12 small sampling areas (S1–S12) were strategically positioned along predefined directional arrows (Figure 10), with S1 to S9 located on Transect A and S10 to S12 on Transect B. These samples were situated in areas of minimal anthropogenic disturbance to represent typical *K. compacta* habitats. Considering environmental heterogeneity, vegetation distribution, topography, and population density, plot dimensions were intentionally varied to capture the species’ key ecological attributes: S1 measured 55 m × 50 m; S2, S4, and S10 were 80 m × 70 m; S3, S5, and S11 were 30 m × 30 m; S6 (15 m × 15 m) was scaled to fully cover community structures constrained by local terrain; S7, S9, and S12 were 65 m × 65 m, 60 m × 50 m, and 70 m × 70 m, respectively; and S8 spanned 100 m × 100 m.

#### 4.1.4. UAV Image Processing

**Figure 11:**
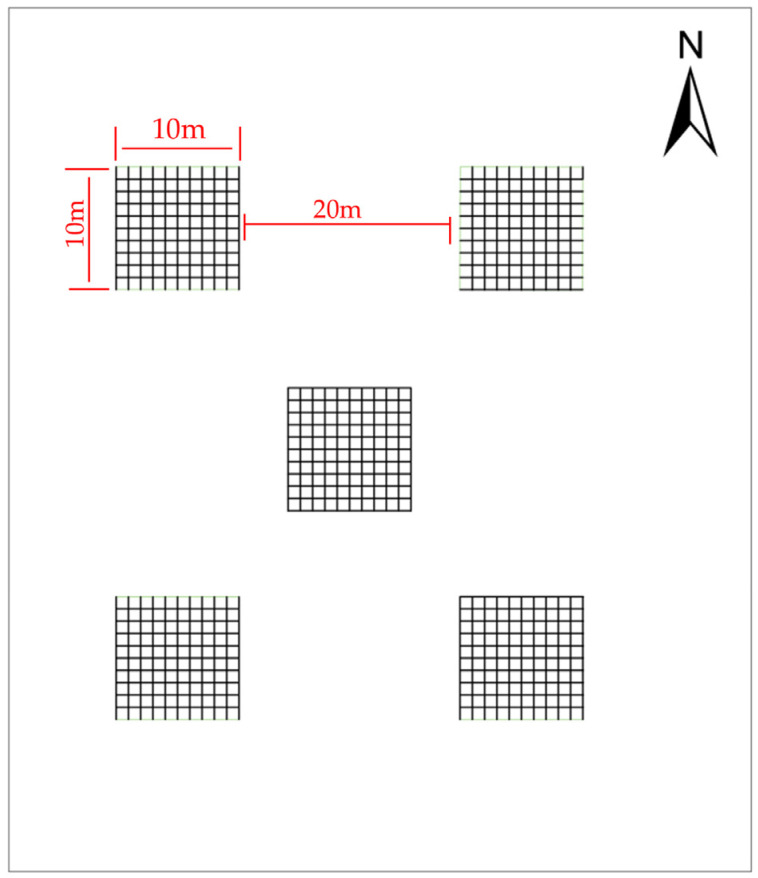
Sampling grid layout in S8.

A DJI Phantom 4 Pro drone was deployed at a flight altitude of 150 m with a 5-second capture interval to acquire multispectral imagery. The images were processed in Agisoft Metashape (v2.1.2) to generate digital orthophoto maps (DOM) and digital surface models (DSM). Given the low stature of *K. compacta*, plant height and crown width measurements were conducted on the ArcGIS 10.8 platform by integrating DOM and DSM data. Each sample was divided into five large grids aligned with cardinal directions. Depending on sample size, each large grid was further subdivided into 1 m × 1 m subgrids: 45 subgrids in S6, 245 in S3, S5, and S11 and 500 in the remaining sample. Within each subgrid, individual *K. compacta* plants were measured (Figure 11). The sampling plots were configured with distinct grid specifications: Plot S6 featured 3 m×3 m grids with 5 m diagonal distance between adjacent grid vertices (measuring corner-to-corner distance), while plots S3, S5, and S11 contained 7 m × 7 m grids spaced at 10 m intervals. The remaining plots were established with 10 m × 10 m grids maintaining 20 m inter-transect spacing. The total sample sizes per plot were as follows: S1 and S2 (152 each), S3 (135), S4 (42), S5 and S6 (17 each), S7 (33), S8 (40), S9 (14), S10 (37), S11 (19), and S12 (49), yielding an initial dataset of 707 plants. Outlier detection in SPSS Statistics 25.0 removed 21 plants (4 from S1 and S2 each, 9 from S3, 1 from S9, 2 from S10, and 1 from S11), resulting in 686 valid plants.

**Figure 12:**
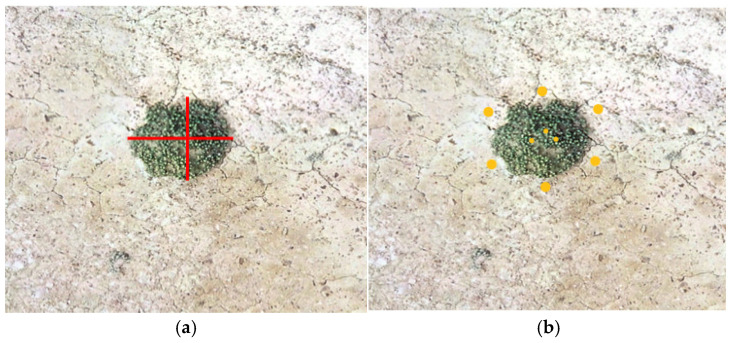
Morphological measurement measurements derived from DOM and DSM. (**a**) Crown diameter measurement; (**b**) Plant height measurement. Note: The red line segments in the left panel represent crown diameter measurements along the east-west and north-south directions, while the yellow points in the right panel denote elevation data extracted for bare ground and the centroid of the crown.

Plant height was calculated as the vertical difference between the mean elevation of the crown center (averaged from three repeated measurements) and the mean elevation of six surrounding bare-ground points. Crown diameter was determined by averaging three repeated measurements of the east-west and north-south diameters, followed by a secondary mean calculation (Figure 12a,b). Radial bar plots illustrating mean plant height, crown diameter, and elevation correlations were generated in Origin 2021.

#### 4.1.5. Point Pattern Data Processing

To mitigate edge effects, subplots were established within the central zones of each main plot using ArcGIS 10.8 for analyzing the spatial distribution of K. compacta. Subplots s1 to s12 (aligned with parent sample S1 to S12) were configured with the following dimensions: s5 (13 m × 13 m), s6 (8 m × 8 m), s8 (35 m × 35 m), and other subplots (25 m × 25 m). Using a neighboring grid approach, each subplot was subdivided into 1 m × 1 m grids: s5 contained 169 grids, s6 had 64 grids, s8 comprised 1225 grids, and other subplots included 625 grids. *K. compacta* individuals were identified via visual interpretation. The lower-left corner of the first grid served as the coordinate origin (0,0), with the center coordinates of each cushion plant recorded to generate 2D spatial datasets for subsequent point pattern analysis.

#### 4.1.6. MaxEnt Model Construction and Validation

To reduce the clustering effect error in the *K. compacta* distribution data, spatial thinning was performed using ENMTools, ensuring only one occurrence point per 30′ × 30′ grid cell, resulting in 14 valid distribution points. The distribution data and current climatic variables were imported into MaxEnt 3.4.4 for modeling, with 75% of the records as the training set and 25% as the test set, replicated five times. The mean of five runs was adopted as the final result. Parameters included 5000 background points, jackknife analysis, and logistic output in .asc format.

**Figure 13:**
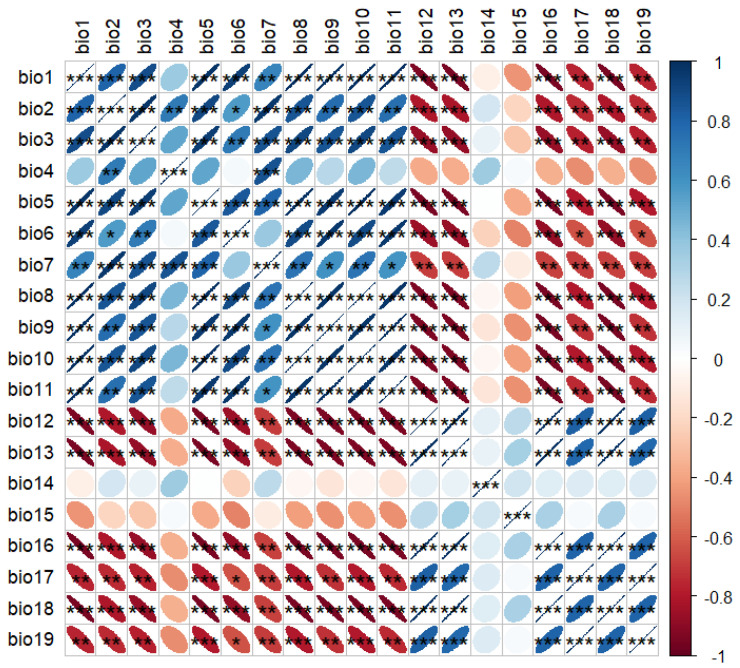
Correlation matrix of the 19 bioclimatic variables (* *p* < 0.05, ** *p* < 0.01, *** *p* < 0.001).

To avoid overfitting under MaxEnt’s default settings, the ENMeval package in R 4.4.1 was employed to optimize the model by adjusting feature combinations (FC) and regularization multipliers (RM). MaxEnt provides five feature classes: Linear (L), Quadratic (Q), Product (P), Threshold (T), and Hinge (H). Six FCs were tested: H (Hinge), L (Linear), LQ (Linear + Quadratic), LQH (Linear + Quadratic + Hinge), LQHP (Linear + Quadratic + Hinge + Product), and LQHPT (Linear + Quadratic + Hinge + Product + Threshold). RM values ranged from 0.5 to 3 (intervals of 0.5), with optimal parameters selected when ΔAICc = 0 [49]. To mitigate multicollinearity, 19 bioclimatic variables were analyzed in R 4.4.1. Variables with |r| > 0.8 and lower contribution (based on jackknife tests) were excluded (Figure 13), retaining bio4, bio7, bio12, bio15, and bio17 (Table 7). The MaxEnt output was imported into ArcGIS 10.8. Reclassification thresholds were determined using the balance training omission, predicted area, and threshold value (TPT) and maximum training sensitivity plus specificity (MTSS) to categorize unsuitable habitat, sub-suitable habitat, and highsuitable habitat [50].

### 4.2. Methods

#### 4.2.1. Plasticity Index

The plasticity index (PI) evaluates the adaptability of plant morphological traits to environmental conditions [28], with higher values indicating greater susceptibility to environmental heterogeneity.

(1) PI=(Xmax-Xmin)/Xmax

In the formula, *X*max denotes the maximum value, and *X*min denotes the minimum value.

#### 4.2.2. Redundancy Analysis

RDA elucidates the relationship between the response variable matrix (e.g., species data) and the explanatory variable matrix (e.g., environmental factors) by projecting their associations onto a low-dimensional orthogonal ordination space [51]. The angular relationships between vectors in the ordination diagram indicate correlations: acute angles (<90°) denote positive correlations between species and environmental factors, obtuse angles (>90°) reflect negative correlations, and right angles (≈90°) suggest no significant association. Furthermore, the length of arrows representing environmental variables corresponds to their explanatory power in shaping species distributions, with longer arrows indicating stronger contributions to the observed variance.

To select the appropriate ordination method, a detrended correspondence analysis (DCA) was first performed on the species matrix. The maximum axis length of the DCA was less than 3, indicating that RDA was suitable. To mitigate the effects of collinearity among predictors, variance inflation factor (VIF) was used to test multicollinearity, and factors with VIF values ≥ 10—including elevation, precipitation, potential evapotranspiration, aridity index, geomorphological type, silt, clay, and soil pH—were excluded. Plant height, crown width, and the remaining six environmental variables were standardized (using Hellinger transformation to shape variable and z-score normalization to the environment variable) prior to analysis. The results were then subjected to Monte Carlo permutation tests (999 permutations, significance threshold: *p* < 0.05) to evaluate the overall model significance and the significance of individual environmental variables. VIF calculation and RDA were conducted in R version 4.4.1 using the car and vegan packages.

#### 4.2.3. Point Pattern Analysis

The univariate pair correlation function *g*(*r*), derived from Ripley’s *K*-function, addresses the inherent limitation of cumulative spatial pattern effects across scales in the *K*-function, where larger-scale patterns are confounded by the aggregation of smaller-scale interactions. To mitigate this bias, we applied the pair correlation function *g*(*r*) for analyzing the spatial point patterns of *K. compacta* along elevation gradients, as formally expressed in Equation (2). This approach isolates spatial associations at specific distances, enabling a scale-specific interpretation of clustering or dispersion independent of cumulative smaller-scale effects.

(2) $$g(r)={\left(2\pi r\right)}^{-1}dK(r)/dr$$

where d*K*(*r*) denotes the differential of the function *K*(*r*), and d*r* represents the differential of the radius r.

The spatial distribution patterns can be determined based on the values of *g*(*r*), typically employing Monte Carlo simulations to generate upper and lower confidence envelopes. If *g*(*r*) lies above the envelopes, it indicates a clustered distribution; between the envelopes, a random distribution; and below the envelopes, a uniform distribution. However, in point pattern analysis, the *g*(*r*) function may exhibit non-aggregative behavior, necessitating the use of null models to quantify deviations between observed and theoretical spatial processes [52]. Common null models include the CSR model, which assumes no biological or abiotic constraints on spatial distribution (i.e., equal and independent occurrence probabilities across all scales), and the heterogeneous Poisson (HP) process, which accounts for habitat heterogeneity by allowing population density to vary with environmental gradients. Analyses were conducted in R 4.4.1 using the spatstat packages, generating 95% confidence envelopes to assess significance, with a step size of 0.05 m. For the HP model, the optimal bandwidth (sigma) was determined using Scott’s rule to adaptively refine spatial smoothing based on local point density.

#### 4.2.4. Correlation Analysis

To explore the relationship between individual morphological variations of *K. compacta* and environmental factors, we applied Pearson correlation analysis. The Pearson correlation coefficient (PCC), a statistical method quantifying the linear association between two variables, is calculated as the ratio of the covariance between the dependent (response) and independent variables to the product of their standard deviations. The coefficient r ranges from −1 to 1: a value of +1 signifies a perfect positive linear correlation, −1 indicates a perfect negative linear correlation, and 0 denotes no linear association [53]. Critically, the PCC is invariant under linear transformations (e.g., unit scaling), ensuring the validity of r values even when variables are measured in different units. This property allows robust cross-comparisons of morphological traits and environmental drivers across heterogeneous datasets. Pearson correlation analysis and heatmap visualization were conducted in R version 4.4.1 using the ggplot2 and corrplot packages.

(3) $$r_{xy}=\frac{\Sigma (x_{i}\;-\;\bar{x})\Sigma (y_{i}\;-\;\bar{y})}{\sqrt{{\Sigma (x_{i}\;-\;\bar{x})}^{2}}\sqrt{{\Sigma (y_{i}\;-\;\bar{y})}^{2}}}$$

where $\bar{x}$ denotes the mean of *x*, and $\bar{y}$ denotes the mean of *y*.

#### 4.2.5. MaxEnt Model

The MaxEnt model can provide accurate predictions for macroecological patterns using limited state variable information [54]. As a general machine learning method, its principle is to identify the probability distribution with maximum entropy under incomplete environmental constraints of species occurrence data, thereby estimating the species’ geographic distribution [55]. The MaxEnt software (v3.4.4), developed by Phillips et al., integrates the maximum entropy principle to predict current and future potential species distributions using occurrence records and bioclimatic variables. Model performance is evaluated via the receiver operating characteristic (ROC) curve, where the area under the ROC curve (AUC) quantifies accuracy, ranging from [0, 1]—higher values indicate better model performance. Generally, 0.5 ≤ AUC < 0.7 signifies poor prediction; 0.7 ≤ AUC < 0.9 indicates acceptable performance; and AUC ≥ 0.9 reflects excellent predictive reliability [10].

## 5. Conclusions

To investigate the morphological variations, spatial distribution patterns, and future suitable habitats of *K. compacta* in the Tibetan antelope breeding grounds of the Western Kunlun Mountains, this study employed Pearson correlation and redundancy analysis (RDA) to analyze morphological changes and their driving forces. Complete spatial randomness (CSR) and heterogeneous Poisson (HP) models were applied to assess spatial distribution, while the MaxEnt model optimized with R was used to predict changes in suitable habitats under future climate scenarios. The results showed that Pearson correlation analysis revealed that crown diameter was positively correlated with elevation, precipitation, and relative humidity but negatively correlated with mean annual temperature, aridity index, and potential evapotranspiration. Plant height exhibited positive correlations with soil pH, mean annual temperature, and potential evapotranspiration but negative correlations with elevation and soil texture types. RDA identified mean annual temperature as the dominant factor driving variations in plant height and crown diameter. Analysis using the two null models indicated that the CSR model demonstrated low distribution density of *K. compacta* across different elevation gradients, with a general pattern of uniform-random-clustered-random distribution. The HP model highlighted that habitat heterogeneity significantly influenced distribution as spatial scale increased. The MaxEnt model projected a notable reduction in suitable habitats for *K. compacta* under the SSP126 pathway during 2061 to the 2080s, whereas the SSP585 pathway suggested an expansion of suitable areas, indicating that a certain degree of warming promotes the expansion of *K. compacta* habitats.

## Figures and Tables

**Table 1:** Descriptive statistics of crown diameter characteristics for *Krascheninnikovia compacta* (Losinsk.) Grubov.

Sampling Areas	Mean/cm	Max/cm	Min/cm	Kurtosis	Skewness	Standard Deviation	CV/%	PI
S1	15.79	18.81	10.98	−0.95	0.002	1.79	11.35	0.42
S2	24.95	35.17	16.67	−0.85	0.40	4.94	19.80	0.53
S3	31.39	44.1	19.22	0.45	−0.01	5.20	16.56	0.56
S4	18.29	20.06	13.31	2.91	−1.75	1.77	9.68	0.34
S5	17.30	24.76	9.45	−0.45	0.04	3.27	18.93	0.62
S6	17.57	23.47	13.46	−0.97	0.52	3.17	18.05	0.43
S7	18.55	27.11	12.58	−0.34	0.44	3.53	19.01	0.54
S8	16.05	23.78	9.86	−0.13	0.55	2.90	18.06	0.59
S9	15.98	25.96	8.13	−0.45	0.33	3.65	22.85	0.69
S10	26.36	37.85	13.39	−1.33	0.19	8.26	31.35	0.65
S11	23.71	36.54	15.67	−0.06	0.68	5.28	22.27	0.57
S12	19.29	25.40	10.78	−0.36	−0.14	3.51	18.22	0.58

**Table 2:** Descriptive statistics of plant height characteristics for *K. compacta*.

Sampling Areas	Mean/cm	Max/cm	Min/cm	Kurtosis	Skewness	Standard Deviation	CV/%	PI
S1	3.44	4.30	2.07	−0.18	−0.35	0.61	17.73	0.52
S2	3.07	4.29	2.03	−0.24	0.28	0.53	17.30	0.53
S3	2.58	3.90	1.47	0.41	0.21	0.52	20.29	0.62
S4	2.95	3.86	1.67	0.19	−0.54	0.65	22.08	0.57
S5	2.63	3.97	1.53	−0.38	0.51	0.04	20.10	0.61
S6	2.73	3.50	2.02	−1.04	−0.26	0.47	17.21	0.42
S7	2.87	3.82	1.67	0.19	0.15	0.51	17.88	0.46
S8	3.07	4.80	1.27	−0.31	0.09	0.70	22.63	0.74
S9	3.00	4.38	153	0.18	0.02	0.54	17.97	0.65
S10	3.39	4.90	1.11	1.87	−0.82	0.95	28.09	0.77
S11	1.30	2.38	0.5	−0.44	0.53	0.49	37.30	0.79
S12	2.02	2.97	1.33	0.31	0.51	0.37	18.32	0.55

**Table 3:** Geographical locations of sampling areas.

Sampling Areas	Longitudes of Site Center (E)	Latitudes of Site Center (N)	Elevations/m
S1	83°19′19.596″	36°25′3.475″	4184
S2	83°0′56.79″	36°15′3.658″	4710
S3	82°54′42.178″	36°13′3.626″	5006
S4	82°32′10.018″	36°5′40.945″	4566
S5	82°29′14.964″	36°3′52.103″	4599
S6	82°24′25.654″	35°58′9.746″	4855
S7	82°23′35.441″	35°57′38.902″	4820
S8	82°22′45.217″	35°57′1.804″	4678
S9	82°21′49.165″	35°56′19.036″	4548
S10	83°2′46.824″	36°6′14.551″	4903
S11	83°0′37.447″	35°56′58.085″	5124
S12	82°56′44.794″	35°48′54.158″	5106

**Table 4:** Coefficient of determination (R^2^) for environmental variables in RDA.

Environmental Factors	RDA1	RDA2	R^2^	Pr(>r)
Slope	−0.94124	0.33773	0.1793	0.406
MAT	−0.98336	0.18166	0.8120	0.001 ***
Texcls	0.99999	0.00452	0.4965	0.085
RH	0.94193	−0.33581	0.5237	0.051
SAND	0.99984	−0.01762	0.2308	0.296
Light	0.98632	−0.16486	0.3276	0.186

Note: *** *p* < 0.001.

**Table 5:** Change in potential suitable area of *K. compacta*.

Period	Suitable Habitat/10^4^ km^2^		
	Highly Suitable Habitat	Sub-Suitable Habitat	Unsuitable Habitat
1970 to 2000s	0.43	0.65	0.39
2061 to 2080s (SSP126)	0.2	0.44	0.83
2061 to 2080s (SSP585)	0.44	0.81	0.21

**Table 6:** Summary of the range change statistics for *K. compacta* under climate change scenarios compared to current climatic conditions.

Climatic Period	Area/10^4^ km^2^			Proportion of Area/%		
	Expansion	Unchanged	Contraction	Expansion	Unchanged	Contraction
Current-2061 to 2080s (SSP126)	0.0016	0.63	0.44	0.1	43.21	30.26
Current-2061 to 2080s (SSP585)	0.2	1.06	0.01	13.4	72.46	1

**Table 7:** Climate and environment variables.

Bioclimatic Variable	Code	Unit
bio1	Annual Mean Temperature	°C
bio2	Mean Diurnal Range (Mean of monthly (max temp—min temp)	°C
bio3	Isothermality (bio2/bio7) (×100)	%
bio4	Temperature Seasonality (standard deviation × 100)	°C
bio5	Max Temperature of Warmest Month	°C
bio6	Min Temperature of Coldest Month	°C
bio7	Temperature Annual Range (bio5-bio6)	°C
bio8	Mean Temperature of Wettest Quarter	°C
bio9	Mean Temperature of Driest Quarter	°C
bio10	Mean Temperature of Warmest Quarter	°C
bio11	Mean Temperature of Coldest Quarter	°C
bio12	Annual Precipitation	mm
bio13	Precipitation of Wettest Month	mm
bio14	Precipitation of Driest Month	mm
bio15	Precipitation Seasonality (Coefficient of Variation)	%
bio16	Precipitation of Wettest Quarter	mm
bio17	Precipitation of Driest Quarter	mm
bio18	Precipitation of Warmest Quarter	mm
Bio19	Precipitation of Coldest Quarter	mm

## Data Availability

Data are contained within the article.

## Abbreviations

The following abbreviations are used in this manuscript:

UAV	unmanned aerial vehicle
DOM	digital orthophoto map
DSM	digital surface model
DEM	digital elevation model
CSR	complete spatial randomness
HP	heterogeneous Poisson
PI	plasticity index
RDA	redundancy analysis
DCA	detrended correspondence analysis
MAT	mean annual temperature
PET	potential evapotranspiration
AI	aridity index
RH	relative humidity
CV	coefficient of variation
VIF	variance inflation factor
PCC	Pearson correlation coefficient
MaxEnt	maximum entropy
ROC	operating characteristic curve
TPT	balance training omission, predicted area, and threshold value
MTSS	maximum training sensitivity plus specificity
